# A Cross‐Sectional Study of the Association of Neck Circumference and Cardiovascular Risk Among Market Women in Ghana

**DOI:** 10.1002/hsr2.71141

**Published:** 2025-08-10

**Authors:** Bertha Sena Odoom, Marina Aferiba Tandoh, Andrews Baah

**Affiliations:** ^1^ Department of Biochemistry and Biotechnology (Human Nutrition) College of Science, KNUST, PMB Kumasi Ghana; ^2^ Department of Biochemistry and Biotechnology (Human Nutrition and Dietetics) College of Science, KNUST, PMB Kumasi Ghana; ^3^ Department of Biochemistry and Biotechnology College of Science, KNUST, PMB Kumasi Ghana

**Keywords:** cardiovascular risk, dietary patterns, hypertension, market women, neck circumference

## Abstract

**Background:**

Cardiovascular diseases (CVDs) are the leading cause of global mortality, with hypertension being a major concern. Majority of CVD‐related deaths occur in developing countries.

**Aim:**

This study examined the relationship between various anthropometric indices, including neck circumference (NC), body mass index (BMI), hip index (HI), waist circumference (WC) and body roundness index (BRI) and hypertension among market women in Koforidua, Ghana.

**Methods:**

A cross‐sectional study involving 175 market women aged 18–60 years was conducted. Data on sociodemographics, dietary patterns, physical activity, anthropometry, and blood pressure were collected. Logistic regression was used to assess associations, and receiver operating characteristic (ROC) analysis evaluated the predictive ability of each index.

**Results:**

The prevalence of hypertension was 53.7%. Age and BMI showed a significant positive correlation with systolic blood pressure (SBP) (*p* < 0.001), while NC was also significantly associated (*p* = 0.019). Weight correlated positively with diastolic blood pressure (DBP) (*p* < 0.05), whereas height correlated significantly negatively (*p* < 0.05). NC was significantly associated with SBP (*β* = 1.185, *p* = 0.015), while BMI was a positive but nonsignificant predictor (*β* = 0.272, *p* = 0.082). ROC analysis identified BMI, NC, and HI as key predictors of hypertension, with BMI (AUC = 0.543 for SBP, 0.646 for DBP) and NC (AUC = 0.504 for SBP) demonstrating notable predictive abilities. Optimal cutoff points for hypertension included 27.65 kg/m² for BMI and 30.50 cm for NC.

**Conclusion:**

NC emerged as a strong predictor of SBP, highlighting its potential as a simple, non‐invasive marker for hypertension. While BMI also showed predictive value, WC and WHR were less relevant. Targeted interventions are needed to mitigate CVD risk among market women.

## 1 Introduction

Cardiovascular diseases (CVDs) are a group of disorders affecting the heart and blood vessels and are the most common cause of death worldwide. About 17.9 million (31%) of deaths worldwide were attributed to cardiovascular illnesses in 2016 [1]. About 85% of these fatalities were due to acute cardiomyopathy, heart disease, and stroke [1]. Fast food, inactivity, technology, and other aspects of modern civilization modify behavior and lifestyle, which changes health and disease outcomes (Agyei‐Mensah, 2010). Age, smoking, hypertension, diabetes, obesity, poor diet, excessive alcohol use, physical inactivity, dyslipidemia, family history of heart disease, ethnicity/gender, and psychological factors are among the risk factors for CVD [2]. Hypertension is the leading cause of mortality in sub‐Saharan Africa, but is often asymptomatic. Symptoms often precede problems such as eye, heart, and kidney disease [3]. Many patients with hypertension are unaware of their condition, especially in Africa. In 2006, only 33%–34% of people living in rural and urban Ghana were aware of hypertension. Only 16.7%–28% received treatment and only 6.2% reached their target (Agyei‐Mensah & de‐Graft Aikins, 2010). It has been observed that as societies modernize, they experience significant changes in their patterns of health and disease. In spite of the rapid modernization globally, there are limited studies of changes in health and disease among sub‐Saharan African countries. This study therefore sought to examine the relationship between various anthropometric indices such as neck circumference (NC), body mass index (BMI), hip index (HI), waist circumference (WC) and body roundness index (BRI) and hypertension among market women in Koforidua, Ghana. There is a double burden of infectious and chronic disease which constitutes major causes of morbidity and mortality in Ghana. This double burden is divided across social class. Whereas wealthy communities experience higher risk of chronic diseases, the poor communities experience a double burden of infectious, as well as chronic diseases. Urbanization, urban poverty and globalization have been implicated in this transition (Agyei‐Mensah & de‐Graft Aikins, 2010).

Obesity has been associated with an increased risk of type 2 diabetes (T2D), metabolic syndrome, and hypertension, which is also considered as a major determinant of cardiovascular disease and premature death. A relationship has been established between obesity and the development of hypertension and T2D, which indicates that obesity is associated with cardiovascular diseases [4]. Additionally, body weight distribution is considered as a determinant of metabolic syndrome as well as the best predictor of cardiovascular risk. Excessive fat accumulation in the body is associated with an increased risk of disease in certain areas [5]. One particular pathogenic fat depot which is strongly associated with insulin resistance, T2D and ischemic heart disease is the visceral adipose tissue [6]. Waist circumference (WC) is widely used in determining central weight due to its relationship with adipose tissue and is considered one of the metabolic parameters in many medical contexts. Previous research has identified a link between central obesity and the development of cardiovascular disease [7]. Recent studies have also shown that neck circumference (NC) is an anthropometric marker directly related to free fatty acids, insulin resistance, very low density lipoprotein (VLDL) cholesterol, cholesterol and hypertension [8]. In addition, another study has suggested using NC as an additional screening method for cardiovascular risk assessment [9]. The aim of this study was to determine the relationship between the neck circumference, blood pressure and CVD risk variables among female traders at Koforidua in the Eastern Region of Ghana.

## 2 Methods

### 2.1 Study Design, Study Site, and Study Population

This study used a cross‐sectional design. It was carried out at the Koforidua Central Market in the Eastern Region. This study was undertaken in May 2021, with a sample size of 175 participants (market women) between 18 and 60 years of age. A simple random sampling was used to recruit the participants. A cross‐sectional study design was employed for this study because it allows for the prevalence and potential associations between anthropometric indicators and cardiovascular risk factors at one time point, although it does not provide insights to changes over a prolonged period.

### 2.2 Sample Size and Sampling

The study population consisted of 333 market women who were currently selling at the Koforidua. However, the results can be applied to similar populations and can potentially help to guide future studies. Market women were selected using a simple random sampling approach, which was accomplished by voting ‘YES’ or ‘NO’, and a person who chose ‘YES’ was selected for the study. Although 333 questionnaires were distributed, only 175 were fully completed, resulting in a 52.6% response rate. The low completion rate was primarily due to some participants' inability to read and write, the demanding nature of their work, and the lack of follow‐up reminders. The simple random sampling method of this study was warranted, since it was exploratory in nature, and ensured that every market woman had an equal chance of being selected, reducing selection bias and allowing the findings to be generalized to a broader population. The purpose of using simple random sampling was to include individuals actively engaged in market activities with diverse anthropometric profiles and lifestyle characteristics relevant to the study. To strengthen future research and improve response rates, alternative data collection methods should be considered, such as using trained interviewers to administer the questionnaire in the local language through face‐to‐face interviews, particularly for participants with low literacy levels. Semi‐structured questionnaires could also be used to facilitate better engagement and ensure more accurate responses.

### 2.3 Inclusion and Exclusion Criteria

The study included market women currently selling at the Koforidua Market in the Eastern Region of Ghana who consented to participate, were healthy, were not taking any medications, and were free from cardiovascular disease. The exclusion criteria encompassed market women with neck deformities or a history of thyroid disease, dyslipidemia, diabetes, hypertension, or any other medical conditions. Participants who had undergone neck surgery or had structural abnormalities of the neck were also excluded to maintain consistency in measurements. These exclusions were implemented to ensure the reliability and validity of the findings by targeting a representative sample of healthy individuals free from confounding health conditions.

### 2.4 Data Collection

A semi‐structured questionnaire was designed for this study to facilitate the systematic gathering of information while allowing flexible follow‐up questions on participants' sociodemographic characteristics, anthropometric and biochemical parameters, dietary assessment, and physical activity levels. Semi‐structured questions combined both structured and open‐ended elements, allowing for flexibility in responses while maintaining a guided framework. These questions ensure that key topics are covered while also providing respondents with the opportunity to elaborate on their experiences, perspectives, and insights [10].

The questionnaire was divided into five sections. Section A captured the background characteristics of respondents, while Section B focused on anthropometric parameters. Sections C, D, and E covered biochemical parameters, dietary assessment (24‐h dietary recall), as well as physical activity levels respectively. To ensure accuracy and cultural sensitivity in data collection, five data collectors underwent comprehensive training. This training included translating questions into the local language and ensuring consistency by re‐translating responses into English. Data collectors distributed the questionnaires to eligible market women, translating questions as necessary to facilitate understanding. They were specifically trained to preserve the intended meaning of each question during translation and practiced re‐translating responses back into English to maintain consistency and accuracy. This approach, emphasized in global women's health research, helped minimize translation bias. Additionally, role‐playing exercises and mock interviews were incorporated into the training to simulate real‐world scenarios, equipping data collectors with the skills to navigate potential challenges, such as variations in dialects and cultural nuances.

### 2.5 Anthropometric Measurements

Anthropometric data included weight, height, body mass index (BMI), waist circumference (WC), neck circumference (NC) and hip circumference (HC). The weight was measured using an electronic scale (SECA 861; range: 0.05–130 kg; precision: 0.05 kg), while the height was measured in the Frankfort plane with a telescopic stature‐measuring instrument (Jactermac, ZT‐150A). WC was assessed with a nonelastic tape (SECA 200; range: 0–150 cm; precision: 1 mm) at the narrowest part of the torso. NC was measured with participants standing upright, arms relaxed at their sides, and head positioned in the Frankfort horizontal plane. A nonelastic tape (SECA 200; range: 0–150 cm; precision: 1 mm) was placed just below the laryngeal prominence, perpendicular to the long axis of the neck. Hip circumference was measured at the widest part of the buttocks. BMI was calculated as weight (kg) divided by height squared (m²), with participants categorized based on standard WHO classifications: underweight (< 18.5 kg/m²), normal weight (18.5–24.9 kg/m²), overweight (25.0–29.9 kg/m²), and obese (≥ 30.0 kg/m²).

Various anthropometric indices were derived using standard formulas. The Body Shape Index (BSI) was calculated from weight, height, and WC, while the Body Adiposity Index (BAI%) was estimated from hip circumference and height. The Body Roundness Index (BRI) was also assessed using the WC and height, while the Conicity Index (CI) was determined using the WC, weight and height. Also, the Hip Index (HI) was determined using the hip circumference and height, while the waist‐to‐height ratio was determined by dividing the WC by height.

### 2.6 Blood Pressure

The systolic and diastolic blood pressure (SBP and DBP, respectively) were recorded using a validated digital automatic blood pressure monitor (Omron M6, Omron Health Care, Kyoto, Japan), based on the International Protocol of the European Society of Hypertension (Topouchian et al., 2006). Participants were asked to sit quietly for 5 min before measurements were taken on their left arm, which was in the extended position. Two readings were obtained, spaced 1–2 min apart. If the initial two readings showed a difference greater than 5 mmHg, an additional measurement was conducted, and the farthest value was discarded. Average values (in mm Hg) were computed separately for SBP and DBP. Low systolic blood pressure was defined as 120 mmHg or lower, and high diastolic blood pressure was defined as 80 mmHg or higher. The study was pre‐tested among a similar population.

### 2.7 Ethics

This study was approved by the Committee on Research, Publication, and Ethics of the School of Medicine and Dentistry, KNUST, Kumasi (CHRPE/AP/812/22). Permission letters were also obtained from the Mayor, City Health Department, other opinion leaders, and store owners to carry out the study. It was voluntary to participate in this study. Verbal consents were required before participation in the survey, covering the study's objective, potential risks or discomforts, potential advantages, privacy and confidentiality policies, and voluntary participation. Security, privacy, and confidentiality were guaranteed. Since the identities of the respondents were not requested, no specific individual could be identified from the data. The characteristics and aims of the research were explained to the women who participated, including their right to leave at any time.

### 2.8 Data Analysis

This study employed the use of the Statistical Package for the Social Sciences (SPSS) version 27 (IBM Inc., Chicago, IL, United States) to analyze the data. Continuous variables were reported as mean ± standard deviation (SD), while categorical variables were presented as frequencies (%). Furthermore, the normality of the study population was assessed using the Shapiro‐Wilk test, P‐P plot, and histogram. The null hypothesis was rejected for all variables, confirming that they exhibited a normal distribution. To investigate the associations among various anthropometric indicators such as BMI, WC, HC, WHR, NC, BSI, BAI, BRI, CI, and HI with blood pressures (both systolic BP and diastolic BP), Pearson correlations were employed. Further, significant associations were evaluated using linear regression analysis. The ability of BMI, neck circumference, hip index, body roundness index, and waist‐to‐height ratio to predict hypertension (SBP ≥ 140 mmHg and/or DBP ≥ 90 mmHg) was assessed using receiver operating characteristic (ROC) curve analysis. The discriminative power of these anthropometric measures was evaluated based on their area under the curve (AUC) with 95% confidence intervals (CIs). Optimal cutoff values for diagnosing hypertension were determined using the Youden index, and the corresponding specificity and sensitivity were determined. The significance level of the analysis was set at *p* < 0.05.

## 3 Results

### 3.1 Socio‐Demographic Characteristics of Study Participants

Table 1 shows the participants' demographic data. In all, 175 respondents completely filled out the questionnaires, resulting in a response rate of 52.6%. The majority (79.4%) of respondents were reported to be 39 years and above, with an average age and standard deviation of 47.06 ± 10.04 years, respectively. Regarding marital status, the majority of respondents (72.0%) were married, 18.9% were single, and 4.6% were separated. Concerning the level of education, 42.9% of respondents had attained Junior High School education, 30.3% had SHS/Vocational education, and about 13.1% had primary education. Only about 5.7% of respondents had no formal education. In terms of monthly household income, the majority (43.1%) earned between 301 and 600 Ghana Cedis, about 17.1% earned between 0 and 300 Ghana Cedis, while a few (8.6%) earned 601 Ghana Cedis and above.

**Table 1:** Socio‐demographic characteristics of study participants.

Variables	Frequency (*n* = 175)	Percentage (%)
**Age Groups**		
18–24	2	1.1
25–31	16	9.1
32–38	18	10.3
39 and above	139	79.4
**Marital status**		
Divorced	4	2.3
Married	126	72.0
Separated	8	4.6
Single	33	18.9
Widow	4	2.3
**Education level**		
JHS	75	42.9
Nil	10	5.7
Primary	23	13.1
SECTECH	53	30.3
Tertiary	14	8.0
**Income level**		
0–300	30	17.1
301–600	130	74.3
601 and above	15	8.6

### 3.2 Descriptive Statistics of Anthropometric Measures and Cardiovascular Parameters Among Market Women (*N* = 175)

Table 2 presents the descriptive statistics of anthropometric measures and cardiovascular parameters among the study participants. The mean age of the participants was 47.06 ± 10.04 years. The mean weight and height were 82.24 ± 10.95 kg and 125.57 ± 3.54 cm respectively, with an average BMI of 35.72 ± 10.95 kg/m². Waist and hip circumferences were recorded as 91.16 ± 12.23 cm and 95.49 ± 11.85 cm respectively, resulting in a mean waist‐to‐hip ratio of 0.95 ± 0.15. The average neck circumference was 33.29 ± 2.17 cm. Additional indices such as body shape index (0.77 ± 0.13), body adiposity index (51.62% ± 9.23%), and body roundness index (79.47 ± 10.55) were also assessed. The mean conicity index and hip index were 0.08 ± 0.01 and 0.69 ± 0.09 respectively. The waist‐to‐height ratio was 0.74 ± 0.10. Regarding cardiovascular variables, the mean systolic blood pressure (SBP) and diastolic blood pressure (DBP) were 132.33 ± 14.06 mmHg and 80.47 ± 9.75 mmHg respectively, while the mean pulse rate was 77.84 ± 12.83 beats per minute.

**Table 2:** Descriptive statistics of anthropometric measures and cardiovascular parameters among market women (*N* = 175).

Variable	Mean ± SD
	*N* = 175
Age (years)	47.06 ± 10.04
Weight (kg)	82.24 ± 10.95
Height (cm)	125.57 ± 3.54
BMI (kg/m^2^)	35.72 ± 10.95
WC (cm)	91.16 ± 12.23
HC (cm)	95.49 ± 11.85
WHR	0.95 ± 0.15
NC (cm)	33.29 ± 2.17
BSI	0.77 ± 0.13
BAI (%)	51.62 ± 9.23
BRI	79.47 ± 10.55
CI	0.08 ± 0.01
HI	0.69 ± 0.09
Waist‐to‐height ratio	0.74 ± 0.10
SBP (mmHg)	132.33 ± 14.06
DBP (mmHg)	80.47 ± 9.75
PR (beats per minute)	77.84 ± 12.83

*Note:* Mean ±SD (standard deviation) and *p* value estimated as < 0.05 at 95% confidence level.

Abbreviations: BAI, Body Adiposity Index; BMI; Body Mass Index; BRI, Body Roundness Index; BSI, Body Shape Index; CI, Conicity Index; HC, hip circumference; HI, Hip Index; NC, neck circumference; WC; waist circumference; WHR, Waist to hip ratio.

### 3.3 Prevalence of Blood Pressure and Anthropometric Indicators Among Market Women (*N* = 175)

Table 3 presents the distribution of participants based on nutritional status, blood pressure classification, and health‐checking habits. The majority (79.4%) of participants were classified as obese, while 18.3% were overweight, and only 2.3% had a normal BMI. Regarding systolic blood pressure, 28.0% had normal BP, while 25.7% were in the pre‐hypertension range, and 28.0% were classified as having stage I hypertension. Similarly, for diastolic blood pressure, more than half (53.7%) had high blood pressure, 27.4% had normal BP, and 18.9% were pre‐hypertensive. In terms of blood pressure monitoring habits, the majority (71.4%) checked their blood pressure only when they visited the hospital, while 16.0% checked once a month, and 12.6% checked once every 3 months. Voluntary check‐up patterns revealed that 69.1% of participants never engaged in voluntary BP checks, while 26.3% checked once a month, 4.0% once a week, and 0.6% had inconsistent monthly check‐ups.

**Table 3:** Prevalence of blood pressure and anthropometric indicators Among Market Women (*N* = 175).

Variables	Frequency (*n* = 175)	Percentage (%)
**Nutritional status (BMI)**		
Normal	4	2.3
Overweight	32	18.3
Obese	139	79.4
**SBP**		
Normal BP	32	18.3
Pre‐hypertension	49	28.0
Stage I High BP	45	25.7
Hypertension crisis	49	28.0
**DBP**		
Normal BP	94	53.7
Per‐hypertension	48	27.4
Stage I High BP	33	18.9
**Check blood pressure**		
Once a month	28	16.0
Once 3 months	22	12.6
Visit the hospital	125	71.4
**Voluntary check‐up**		
Never	121	69.1
Once a month	47	26.9
Once a week	7	4.0

*Source:* Field data (2023).

Abbreviations: BP, blood pressure; BMI, Body Mass Index.

### 3.4 Dietary Pattern Among Market Women (*N* = 175)

Table 4 presents the dietary patterns of the market women, analyzed across six food groups categorized into low dietary pattern, moderate dietary pattern, and high dietary patterns. Low dietary consumption pattern was derived from foods that were consumed monthly, moderate consumption pattern from foods that was consumed rarely, and weekly and high consumption pattern was derived from foods that were consumed daily, 44.6% and 29.7% of the respondents consumed carbohydrate foods (cereals, bread, other staples, pasta, roots and tubers) moderately and highly respectively. Foods like meat, fish and poultry intake were low (44.6%) among respondents. Dairy and dairy products were moderately consumed by the respondents (48.6%), whiles 12.6% of them highly consumed dairy products. Additionally, soups, stews sauces and staples were highly (47.4%) and moderately (26.9%) consumed by the respondents. However, soda drinks intake were high (33.7%) and moderate (38.3%) among them. Furthermore, about 41.1% and 30.3% of the respondents highly and moderately consumed alcoholic beverages respectively.

**Table 4:** Dietary pattern among market women (*N* = 175).

Variables	Frequency (*n* = 175)	Percentage (%)
**Carbohydrate**		
Low	45	25.7
Moderate	78	44.6
High	52	29.7
**Proteins**		
Low	78	44.6
Moderate	67	38.3
High	30	17.1
**Dairies and dairy products**		
Low	68	38.9
Moderate	85	48.6
High	22	12.6
**Soups, stews sauces and staples**		
Low	45	25.7
Moderate	47	26.9
High	83	47.4
**Soda drinks**		
Low	49	28.0
Moderate	67	38.3
High	59	33.7
**Alcoholic beverages**		
Low	50	28.6
Moderate	72	41.1
High	53	30.3

### 3.5 Dietary Practices Among Study Participants

Dietary practices of respondents were analyzed and the results are shown in Table 5. More than half of the respondents (64.6%) reported skipping breakfast everyday. In terms of salt intake, the majority of respondents (93.1%) added salt to their diet, with about 92.0% adding salted fish during cooking. In addition, 44.0% of the respondents' eating habits indicated that they used natural flavorings for cooking, 15.4% used artificial flavorings, and 39.4% used both natural and artificial flavorings. However, only 1.1% of respondents did not use natural or artificial fragrances. In addition, about 12.0% of the respondents ate fruits and vegetables occasionally, and 22.3% ate fruits and vegetables daily. About 13.1% of the respondents never ate fruits and vegetables. However, about half of the people surveyed eat fruit and vegetables every week. In addition, about 60.6% of respondents did not eat sweets, chocolates and cakes, while 22.9% and 13.7% ate sweets, chocolates and cakes, respectively every month and every week. In terms of water consumption, three‐quarters of respondents (73.7%) drank more than 1.25 L of water per day, and 18.9% drank between 501 mL and 1.25 L per day.

**Table 5:** Dietary practice among market women (*N* = 175).

Variables	Frequency (*n* = 175)	Percentage (%)
**Skip breakfast**		
No	62	35.4
Yes	113	64.6
**Add salt to food**		
No	12	6.9
Yes	163	93.1
**Spices used in cooking**		
Artificial	27	15.4
Natural	77	44.0
Both	69	39.4
None	2	1.1
**Salted fish in cooking**		
No	14	8.0
Yes	161	92.0
**Fruit and vegetables intake**		
Never	23	13.1
Occasionally	21	12.0
Per day	39	22.3
Per week	92	52.6
**Sweets, chocolates and pastries**		
Never	106	60.6
Per day	5	2.9
Per month	40	22.9
Per week	24	13.7
**Water intake**		
0–500 mL	13	7.4
501 mL–1.25 L	33	18.9
> 1.25 L	129	73.7

### 3.6 Lifestyle Changes (Physical Activity) Among Market Women (*N* = 175)

Table 6 shows the physical activity levels of the respondents. Self‐reported physical activity data showed that participants met the 60‐min guideline for vigorous, moderate and walking activities. About 6.9% of the respondents practiced vigorous physical activity once within the month, while 11.4% of them practiced vigorous activity once a week. However, more than three quarters of the respondents never practiced any form of vigorous physical activity. Among the respondents who performed some vigorous physical activity, about 14.3% of them spent about an hour during the performance of the physical activity, while only 4.0% of them spent about two (2) hours in performing vigorous physical activity. Additionally, about 5.7% of the respondents performed moderate physical activity for at least 4–7 days within the week. Majority (75.4%) of the respondents never performed moderate physical activity within the week. With respect to the time spent on walking as a form of physical activity, more than three quarters of the respondents (78.3%) walked for an hour almost every day within the week. Furthermore, about 16.6% of the respondents spent about eight (8) hours almost everyday within the week on just walking. However, the respondents spent 21–40 hours sitting within the week, while about 20% the respondents spent 41–60 hours sitting within the week.

**Table 6:** Lifestyle changes (physical activity) among market women (*N* = 175).

Variables	Frequency (*n* = 175)	Percentage (%)
**Vigorous physical activities**		
Never	143	81.7
Once a month	12	6.9
Once a week	20	11.4
**Time spent on vigorous physical activities**		
1 h a day	25	14.3
2 h a day	7	4.0
None	143	81.7
**Moderate physical activities**		
1–3 days	33	18.9
4–7 days	10	5.7
Never	132	75.4
**Time spent on walking**		
1 h a day	137	78.3
2 h a day	9	5.1
8 h a day	29	16.6
**Time spent sitting on weekdays**		
1–20 h	34	19.4
21–40 h	106	60.6
41–60 h	35	20.0
**Sit, stand, or walk during daily activities**		
Sit	125	71.4
Stand	15	8.6
Walk	35	20.0

### 3.7 Correlation Between Anthropometric Measures, and Blood Pressure Values Among Market Women in Ghana

The correlation between anthropometric measures and blood pressure is summarized in Table 7. A significant positive correlation was observed between age and SBP (*p* < 0.001), while no significant correlation was found with DBP (*p* = 0.823). There was a significant positive correlation between weight and DBP (*p* < 0.05) but not with SBP (*p* = 0.086). Height demonstrated a significant negative correlation with DBP (*p* < 0.05), whereas its correlation with SBP was not significant (*p* = 0.244). BMI was significantly correlated with SBP (*p* < 0.001) but not with DBP (*p* = 0.062). NC was significantly correlated with SBP (*p* = 0.019) but not with DBP (*p* = 0.149). Other anthropometric indices, including hip circumference, waist circumference, waist‐to‐hip ratio, conicity index, body shape index and hip index showed varying degrees of association, with waist‐to‐height ratio and body roundness index demonstrating a significant correlation with SBP (*p* = 0.045).

**Table 7:** Correlation between neck circumference, standard anthropometric measures, and blood pressure values among market women in Ghana.

Variables	SBP (mmHg)	DBP (mmHg)
Age (years)	*r* = 0.206	*r* = 0.017
	*p* < 0.001	*p* = 0.823
Weight (kg)	*r* = 0.130	*r* = 0.176
	*p* = 0.086	*p* < 0.05
Height (cm)	*r* = −0.089	*r* = −0.167
	*p* = 0.244	*p* < 0.05
Body Mass Index (kg/m^**2**^ **)**	*r* = 0.141	*r* = 0.244
	*p* = 0.062	*p* < 0.001
Neck circumference (cm)	*r* = 0.177	*r* = 0.110
	*p* = 0.019	*p* = 0.149
Waist circumference (cm)	*r* = 0.016	*r* = 0.123
	*p* = 0.838	*p* = 0.105
Hip circumference (cm)	*r* = −0.091	*r* = 0.138
	*p* = 0.231	*p* = 0.068
Waist‐to‐hip ratio	*r* = 0.068	*r* = 0.025
	*p* = 0.374	*p* = 0.742
Body shape index	*r* = −0.122	*r* = −0.097
	*p* = 0.107	*p* = 0.201
Body roundness index	*r* = −0.056	*r* = 0.151
	*p* = 0.459	*p* = 0.045
Conicity index	*r* = −0.040	*r* = 0.053
	*p* = 0.596	*p* = 0.489
Hip index	*r* = −0.056	*r* = 0.175
	*p* = 0.464	*p* = 0.020
Waist to height ratio	*r* = 0.029	*r* = 0.152
	*p* = 0.701	*p* = 0.045

Abbreviations: BAI, Body Adiposity Index; BMI, Body Mass Index; BRI, Body Roundness Index; BSI, Body Shape Index; CI, Conicity Index; DBP, diastolic blood pressure; HC, hip circumference; HI, Hip Index r‐ Pearson's correlation coefficient; NC, neck circumference; SBP, systolic blood pressure; WC, waist circumference; WHR, waist to hip ratio.

### 3.8 Association of Neck Circumference, Body Mass Index, Body Roundness Index, Hip Index, and Waist‐to‐Height Ratio With Systolic Blood Pressure

Table 8 summarizes the associations between various body composition indices and systolic blood pressure (SBP). Neck circumference was significantly associated with SBP (*β* = 1.185, *p* = 0.015). Although BMI predicted of SBP (*β* = 0.272, *p* = 0.082), the association was not statistically significant. There was a significant association between age and SBP (*β* = 0.257, *p* = 0.015), while hip index and waist‐to‐height ratio were negatively associated but not significant. Body roundness index showed a positive association (*β* = 5.296, *p* = 0.083).

**Table 8:** Regression analysis of the association between anthropometric indices and systolic blood pressure.

SBP			
Predictor	*β* (B)	*t*	95% CI (Lower to Upper)
Model (Constant)	22.291 (62.224)**	2.791	18.217 to 106.231
Age	0.104 (0.257)**	2.459	0.051 to 0.463
Body Mass Index	0.156 (0.272)	1.748	0.035 to 0.579
Neck Circumference (NC)	0.481 (1.185)**	2.461	0.234 to 2.135
Body Roundness Index	3.039 (5.296)	1.743	−0.704 to 11.297
Hip Index	13.301 (−20.963)	−1.576	−47.221 to 5.295
Waist‐to‐height ratio	310.440 (−537.888)	−1.733	−1150.754 to 74.977

*Note:* Significant associations (*p* < 0.05) were observed for age and neck circumference (NC). Β: standardized coefficients, B: unstandardized coefficients, and CI: 95% confidence interval. **p < 0.05; **p < 0.01.

### 3.9 Association of Body Mass Index, Hip Index, Weight and Height With Diastolic Blood Pressure

Table 9 summarizes the associations between various body composition indices and DBP. Body mass index was positively associated but did not reach statistical significance (*β* = 0.843, *p* = 0.100). Similarly, hip index showed a positive association (*β* = 14.023, *p* = 0.099) but was not significant. Weight and height were not significantly associated with DBP.

**Table 9:** Association between anthropometric indices and systolic blood pressure: a regression analysis.

DBP			
Predictor	*β* (B)	*t*	95% CI (lower to upper)
Model (Constant)	84.911 (−34.093)	−0.402	−201.709 to 133.523
Body Mass Index	0.510 (0.843)	1.653	−0.164 to 1.849
Hip Index	8.444 (14.023)	1.661	−2.646 to 30.692
Weight (Kg)	0.232 (−0.239)	−1.031	−0.697 to 0.219
Height (cm)	0.673 (0.764)	1.135	−0.564 to 2.091

*Note:* No significant associations were observed (*p* > 0.05). β: standardized coefficients, B: unstandardized coefficients, and CI: the 95% confidence interval.

### 3.10 ROC Analysis of Age, BMI, NC, BRI, Hip Index, and WHR for the Prediction of Hypertension

**Figure 1:**
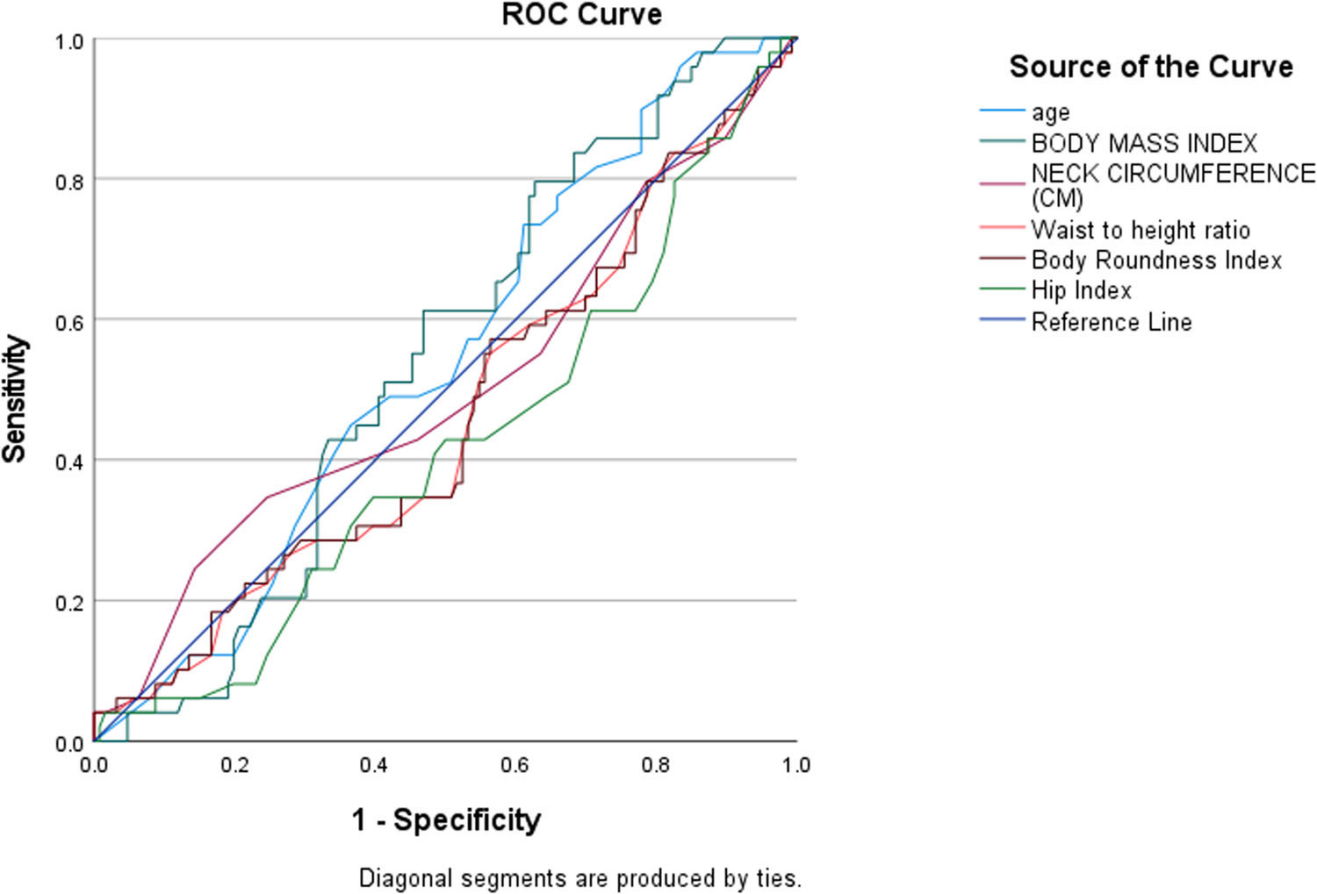
The receiver operating characteristic (ROC) curve for anthropometric predictors of hypertension.

Figure 1 represents the Receiver Operating Characteristic (ROC) curves of age, BMI, Neck Circumference (NC), Body Roundness Index (BRI), Hip Index, and Waist‐to‐Height Ratio (WHR) for predicting systolic blood pressure (SBP) classification. The discriminative abilities of these variables were assessed using the AUC. The AUC values for each predictor were as follows: age (AUC = 0.541, 95% CI: 0.631–0.452), BMI (AUC = 0.543, 95% CI: 0.631–0.456), NC (AUC = 0.504, 95% CI: 0.605–0.403), BRI (AUC = 0.463, 95% CI: 0.559–0.367), Hip Index (AUC = 0.419, 95% CI: 0.512–0.325), and WHR (AUC = 0.461, 95% CI: 0.5570.366). The optimal cutoff points for predicting hypertension based on SBP were 33.50 (sensitivity = 0.959, specificity = 0.167) for age, 27.65 (sensitivity = 1.00, specificity = 0.897) for BMI, 30.50 cm (sensitivity = 0.857, specificity = 0.897) for NC, 107.5500 (sensitivity = 0.410, specificity = 1.00) for BRI, 0.5550 (sensitivity = 0.9590, specificity = 0.9440) for Hip Index, and 1.0200 (sensitivity = 0.041, specificity = 1.000) for WHR.

### 3.11 ROC Curve for Anthropometric Predictors of Hypertension

**Figure 2:**
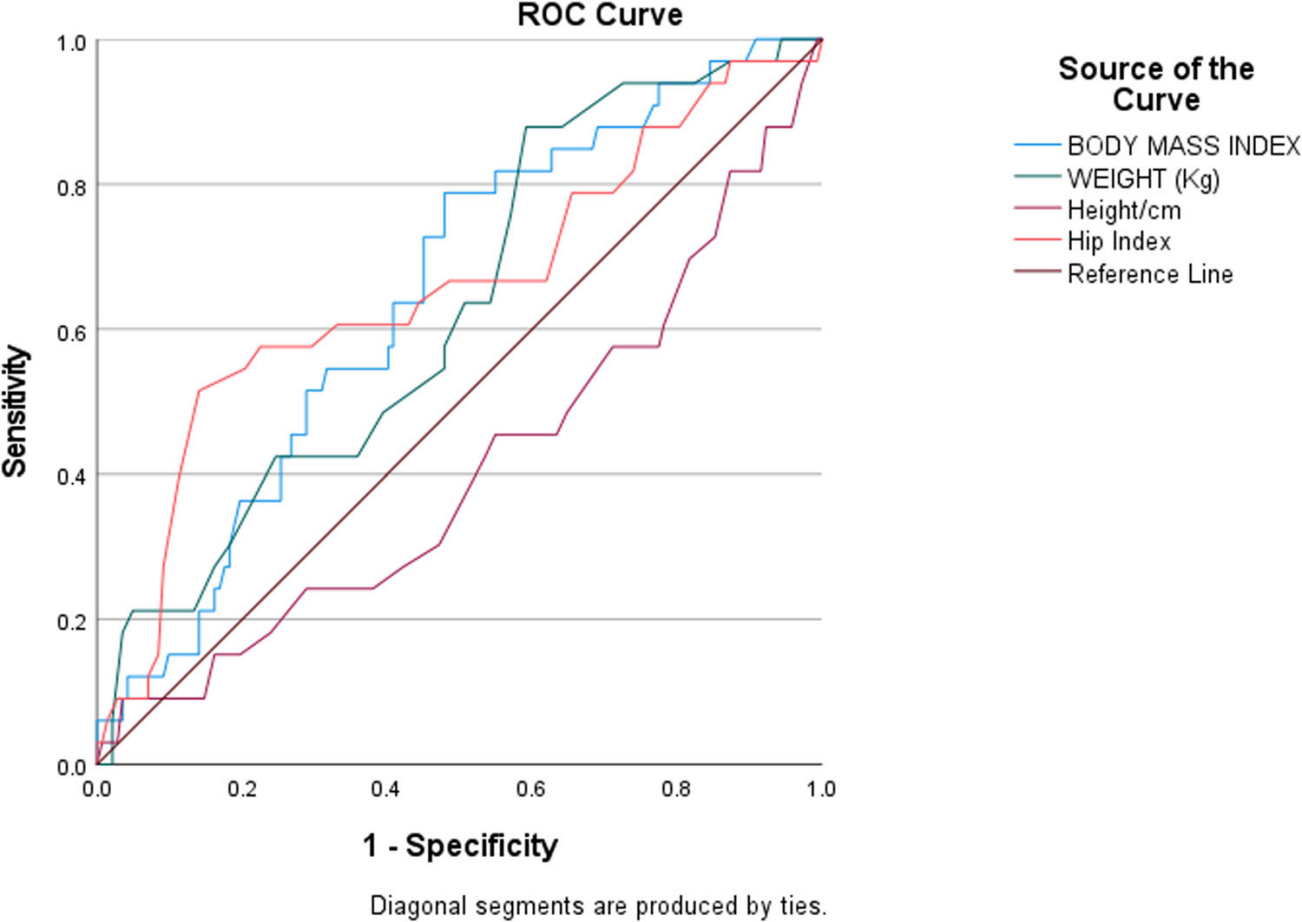
The receiver operating characteristic (ROC) curve for anthropometric predictors of hypertension.

Figure 2 presents the ROC curves of BMI, Weight, Height, and Hip Index for predicting DBP classification. The discriminative abilities of these variables were assessed using the AUC. The AUC values for each predictor were as follows: BMI (AUC = 0.646, 95% CI: 0.548–0.743), Weight (AUC = 0.627, 95% CI: 0.526–0.727), Height (AUC = 0.408, 95% CI: 0.295–0.521), and Hip Index (AUC = 0.657, 95% CI: 0.543–0.770). The optimal cutoff points for predicting hypertension based on SBP were 27.6500 kg/m^2^ (sensitivity = 1.000, specificity = 0.908) for BMI, 68.50 kg (sensitivity = 0.970, specificity = 0.873) for Weight, 116.615 cm (sensitivity = 0.9390, specificity = 0.9720) for Height, and 0.5550 (sensitivity = 0.9700, specificity = 0.9440) for Hip Index.

## 4 Discussion

The prevalence rate of hypertension among market women in this study was 53.7%. Specifically, 28.0% of them had normal systolic blood pressure, 25.7% were prehypertensive, and 28.0% were classified as having stage I hypertension. For diastolic blood pressure, more than half (53.7%) were hypertensive. These findings are consistent with previous studies, such as Bosu and Bosu (2021), who reported a high prevalence of elevated (SBP > 140 mmHg) or (DBP > 90 mmHg) among Ghanaian women. Similarly, Lowe et al. (2015) found that 22% of older women not on anti‐hypertensive medications and approximately 18% of adult women in their study were hypertensive. Moreover, the majority of participants in the present study exhibited poor blood pressure monitoring habits, with 71.4% checking their BP only during hospital visits and 69.1% never engaging in voluntary check‐ups—highlighting a significant gap in preventive health practices.

Lifestyle behaviors play a critical role in hypertension risk. In this study, 64.6% of market women skipped breakfast daily, while 93.1% and 92.0% added salt to food and fish, respectively. High salt intake has been associated with reduced life expectancy, particularly among women [11]. Regarding dietary habits, only 12.0% of the participants consumed fruits and vegetables frequently, and 22.3% consumed them daily. This aligns with a study by Kpodo & Mensah [12], who found that most individuals fail to meet the recommended intake of at least five daily portions. Additionally, 73.7% of the respondents drank over 1.25 L of water daily, which may influence menstrual health [13].

Physical activity levels among market women were generally low to moderate, with many engaging in prolonged walking as part of their occupation. This aligns with studies showing that businesswomen often lack structured physical activity despite its cardiovascular benefits [14]. Obesity remains a significant CVD risk factor, alongside overweight and diabetes [15]. Gender disparities in CVD risk increase with age, particularly among women due to declining estrogen levels [15]. Additionally, access to preventive measures remains unequal across populations [16].

Anthropometric measurements indicated that the average height of market women was 125.57 ± 3.54 cm, and their average body mass index (BMI) was 35.72 ± 10.95 kg/m², classifying most participants as obese. The mean waist circumference (WC) was 91.16 ± 12.23 cm, which is considerably higher than the 31.59 ± 1.18 cm reported in a study by Zanuncio et al. [9]. This discrepancy may be attributed to differences in study populations and measurement techniques. Asosega et al. [17] emphasized the need for effective public health strategies to mitigate weight‐related diseases among women of reproductive age.

A significant positive correlation was observed between BMI and neck circumference (NC) (*r* = 0.172, *p* = 0.023), indicating that as BMI increased, NC also increased. Given that high BMI is associated with cardiovascular diseases (CVDs), including hypertension, NC may also contribute to an increased risk of hypertension. The study also revealed a significant correlation between age and SBP (*r* = 0.206, *p* < 0.001), suggesting that older women had higher SBP levels. However, age was not significantly associated with DBP (*r* = 0.017, *p* = 0.823). Weight was positively associated with DBP (*r* = 0.176, *p* < 0.05), while BMI showed a significant positive correlation with DBP (*r* = 0.244, *p* < 0.001), reinforcing that women with higher BMI tend to have elevated diastolic pressure. NC was also significantly correlated with SBP (*r* = 0.177, *p* = 0.019) but not with DBP (*r* = 0.110, *p* = 0.149), highlighting its potential as an indicator of hypertension risk. Interestingly, WC did not show a significant correlation with either SBP (*r* = 0.016, *p* = 0.838) or DBP (*r* = 0.123, *p* = 0.105), whereas the body roundness index (BRI) and hip index (HI) were significantly correlated with DBP (*p* = 0.045 and *p* = 0.020, respectively). These findings suggest that anthropometric indices, particularly BMI and NC, may serve as predictors of hypertension risk in women. This is consistent with a study by Ramoshaba et al. [18], who reported that an increase in subcutaneous fat, reflected in NC, contributes to hypertension development.

Linear regression analysis further confirmed the associations between anthropometric indices and blood pressure. Age (*β* = 0.104, *p* < 0.05) and NC (*β* = 0.481, *p* < 0.05) were significantly associated with SBP, supporting existing literature that age is a major contributor to systolic hypertension [6]. Additionally, NC demonstrated a strong positive association with SBP, aligning with a study by Preis et al. [8], who suggested that increased NC is linked to higher blood pressure due to its relationship with central obesity and metabolic disturbances. Although BMI (*β* = 0.156, *p* = 0.08) and BRI (*β* = 3.039, *p* = 0.08) showed positive associations with SBP, they were not statistically significant. Linderman et al. [19] analyzed data from 1.7 million adults and found a positive correlation between BMI and both SBP and DBP, supporting the hypothesis of a causal relationship between obesity and hypertension. Similarly, Zhan et al. [20] reported that individuals with higher BRI values had an increased risk of developing hypertension, underscoring BRI's potential as a predictive marker for high blood pressure. Waist‐to‐height ratio (*β* = −1.733, *p* = 0.08) and HI (*β* = −1.576, *p* = 0.12) were not significantly associated with SBP in this study.

In the analysis of DBP predictors, BMI, HI, weight, and height were not significantly associated with DBP, suggesting they may not be strong independent predictors. Although BMI (*β* = 0.510, *p* = 0.10) and HI (*β* = 8.444, *p* = 0.10) exhibited positive associations with DBP, they were not statistically significant. This contrasts with the findings of Van et al. (2025), who reported that overweight and obese individuals were 1.81 and 1.91 times more likely, respectively, to develop hypertension compared to those with normal BMI.

ROC curve analysis provided insights into the predictive power of BMI, NC, BRI, HI, and waist‐to‐height ratio for SBP. BMI exhibited the highest predictive ability (AUC = 0.543), though only slightly above chance, indicating weak discriminatory capacity. Age (AUC = 0.541) showed comparable but limited predictive value. NC (AUC = 0.504) had minimal predictive capability, reinforcing that while central adiposity is linked to hypertension, NC alone may not be a strong independent predictor. Waist‐to‐height ratio (AUC = 0.461) and BRI (AUC = 0.463) had lower predictive values, while HI exhibited the weakest predictive power (AUC = 0.419). These findings suggest that these anthropometric indices have limited utility in SBP classification, possibly due to physiological changes associated with aging [21]. A study among South African adolescents found that BMI had an AUC of 0.621 for SBP, with waist‐to‐height ratio demonstrating slightly better predictive accuracy, suggesting that alternative anthropometric measures may enhance risk classification.

For DBP, ROC curve analysis identified HI as the strongest predictor (AUC = 0.657), followed by BMI (AUC = 0.646), suggesting moderate discriminatory power. Weight also demonstrated some predictive capability, whereas height had the weakest predictive ability (AUC = 0.521).

## 5 Conclusion

This study provides an understanding of using non‐invasive anthropometric indicators for the prediction of hypertension among market women. BMI emerged as the strongest predictor of systolic blood pressure, though its discriminatory power was weak. Neck circumference also demonstrated a significant positive association with SBP, highlighting its potential as an indicator of hypertension risk. However, waist circumference and waist‐to‐height ratio did not show significant correlations with SBP or DBP, suggesting limited predictive value in this population. Hip index demonstrated moderate discriminatory power for DBP classification, while body roundness index showed only a weak association with blood pressure measures. These findings underscore the role of abdominal obesity in hypertension risk but suggest that traditional indices such as BMI and NC may be more relevant in this context. Longitudinal studies are needed to validate these results and to assess the long‐term predictive value of these indices in hypertension prevention and management. Ultimately, these insights could help clinicians develop tailored screening and intervention strategies for such populations based on simple, noninvasive anthropometric measures.

## 6 Limitations

The study was a community—based one and excluded those who were absent from the market on that day. Many of the respondents relied on their memory to recollect dietary patterns. This could potentially introduce a recall bias to the study, but other studies have proven that dietary recall is a valuable tool for collecting nutrition‐related data at the community level.

## Author Contributions

**Bertha Sena Odoom:** investigation, methodology, project administration, writing – review and editing, data curation, writing – original draft, visualization, resources. **Marina Aferiba Tandoh:** conceptualization, methodology, supervision, writing – original draft, writing – review and editing, resources. **Andrews Baah:** writing – original draft, formal analysis, writing – review and editing, data curation, resources.

## Conflicts of Interest

The authors declare no conflicts of interest.

## Transparency Statement

The lead author Marina Aferiba Tandoh affirms that this manuscript is an honest, accurate, and transparent account of the study being reported; that no important aspects of the study have been omitted; and that any discrepancies from the study as planned (and, if relevant, registered) have been explained.

## Data Availability

The data that support the findings of this study are available from the corresponding author upon reasonable request.
